# Haplotyping interspecific hybrids by dual alignment to both parental genomes

**DOI:** 10.1002/tpg2.20324

**Published:** 2023-04-14

**Authors:** Pat J. Brown

**Affiliations:** ^1^ Department of Plant Sciences University of California Davis California USA

## Abstract

Sequencing‐based genotyping of heterozygous diploids requires sufficient depth to accurately call heterozygous genotypes. In interspecific hybrids, alignment of reads to both parental genomes simultaneously can generate haploid data, potentially eliminating the problem of heterozygosity. Two populations of interspecific hybrid rootstocks of walnut (*Juglans*) and pistachio (*Pistacia*) were genotyped using alignment to the maternal genome, paternal genome, and dual alignment to both genomes simultaneously. Downsampling was used to examine concordance of imputed genotype calls as a function of sequencing depth. Dual alignment resulted in datasets essentially free of heterozygous genotypes, simplifying the identification and removal of cross‐contaminated samples. Concordance between full and downsampled genotype calls was always highest after dual alignment. Nearly all single nucleotide polymorphisms (SNPs) in dual alignment datasets were shared with the corresponding single‐parent datasets, but 60%–90% of single‐parent SNPs were private to that dataset. Private SNPs in single‐parent datasets had higher rates of heterozygosity, lower levels of concordance, and were enriched in fixed differences between parental genomes (“homeo‐SNPs”) compared to shared SNPs in the same dataset. In multi‐parental walnut hybrids, the paternal‐aligned dataset was ineffective at resolving population structure in the maternal parent. Overall, the dual alignment strategy effectively produced phased, haploid data, increasing data quality and reducing cost.

## Core Ideas

- Sequencing‐based genotyping of diploids suffers from undercalling of heterozygous genotypes.
- In interspecific hybrids, dual alignment to both parental genomes results in haploid genotypic datasets.
- Dual alignment reduces the proportion of “homeo‐SNPs” (fixed differences between nonallelic sites).

**Abbreviations**
GBS: genotyping by sequencing
MAF: minor allele frequency
SAM: sequence alignment map
SNP: single nucleotide polymorphism

## 1 INTRODUCTION

The prodigious throughput of short‐read sequencing technology has revolutionized quantitative genetics by allowing multiplexed genome‐wide genotyping of large numbers of individuals with minimal ascertainment bias (Andrews et al., 2016; Davey et al., 2011). A major technical challenge to this approach is accurate calling of heterozygous genotypes at low sequencing depth. To circumvent this problem, reduced‐representation libraries are often generated using restriction enzymes (Baird et al., 2008; Elshire et al., 2011) or sequence capture (Ali et al., 2016; Gnirke et al., 2009), increasing sequencing depth across a subset of the genome. However, haploid or inbred individuals can be genotyped and imputed more accurately and inexpensively than heterozygous individuals (Swarts et al., 2014). A second challenge to genotyping with low‐depth short‐read data is the possibility of “homeo‐SNPs” arising from alignment of reads from homeologous regions of the genome (Hulse‐Kemp et al., 2015; Tinker et al., 2014). A homeo‐SNP can be defined as an apparent single nucleotide polymorphism (SNP) that results from alignment of nonallelic reads. Homeo‐SNPs can arise due to incomplete reference assemblies or structural differences between genomes and are expected to appear heterozygous in all samples with sufficient read depth. These false polymorphisms can often be identified by their excess heterozygosity relative to Hardy–Weinberg equilibrium, but homeo‐SNPs that escape filtering may interfere with imputation and estimation of relatedness between individuals. Homeo‐SNPs are particularly problematic in polyploids and interspecific hybrids.

Polyploidy and interspecific hybridization are common features of plant evolution that are exploited in plant breeding to generate novelty, increase vigor, and stack desirable alleles from different species (Alix et al., 2017). Tree and vine crops often rely on interspecific hybrid rootstocks to increase vigor and resilience to biotic or abiotic stresses without affecting fruit or nut quality in the grafted scion (Warschefsky et al., 2015). In California, for example, production of almonds (*Prunus dulcis*) (Ledbetter & Sisterson, 2008), walnuts (*Juglans regia*) (Ramasamy et al., 2021), and pistachios (*Pistacia vera*) (Ferguson et al., 2002) relies on rootstocks that are interspecific hybrids. Each of these nut crops has a mating system that can be exploited to generate large numbers of hybrid progeny (self‐incompatibility, monoecy, and dioecy, respectively), and superior hybrid genotypes can be propagated clonally. However, genetic gain in rootstock breeding programs is generally slow due to the time and space required for evaluation, as well as the difficulty of genotyping highly heterozygous material.

**FIGURE 1:**
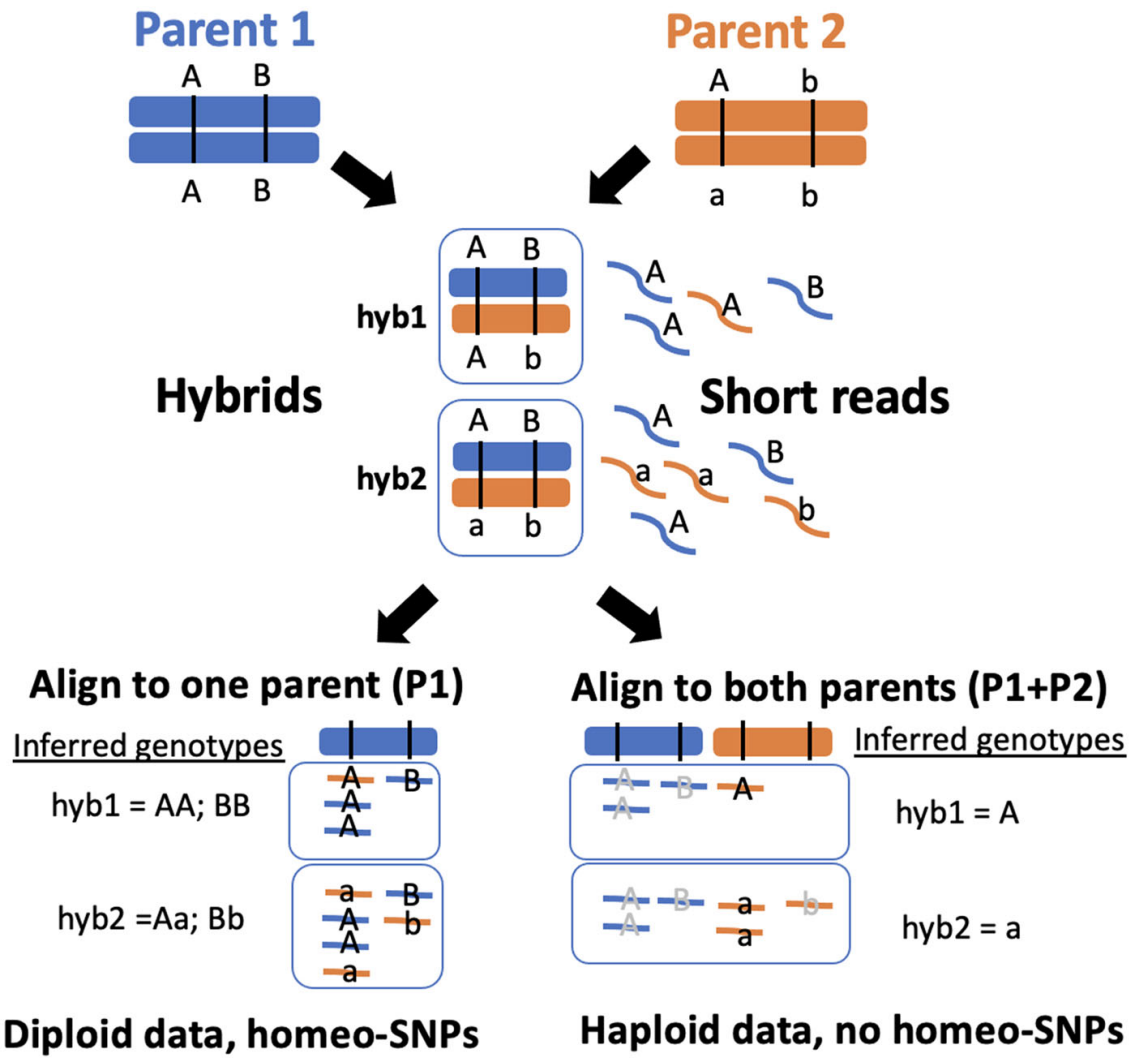
Rationale for dual alignment of sequence reads from interspecific hybrids to both parental genomes. Locus A is truly polymorphic in Parent 2, whereas locus B represents a fixed difference between parents. Although all hybrids are heterozygous at locus B, alignment of low‐depth data to a single parental genome may result in homozygous single nucleotide polymorphism (SNP) calls at this locus (as shown in hyb1), leading to the incorrect calling of a “homeo‐SNP.” Dual alignment to both parental genomes is expected to avoid this problem. Locus A, a true SNP, segregates as a diploid locus (AA versus Aa) when aligning to a single parent, and segregates as a haploid locus (A versus a) when aligning to both parents. Alignment to a single parent requires depth thresholding whereas dual alignment does not.

This study evaluates different methods for generating genotype data from elite populations of interspecific hybrid pistachio (*Pistacia atlantica × Pistacia integerrima*; *n* = 725) and walnut (*Juglans microcarpa × J. regia*; *n* = 228) rootstocks. All parental trees in both genera are highly heterozygous, so the resulting interspecific hybrids are segregating full‐sib families. Short‐read sequencing was performed on reduced‐representation libraries for each species. A typical workflow would be to align the resulting reads against either the maternal (P1) or paternal (P2) genome (Figure 1). Because interspecific hybrids are composed of one haploid gamete from each parent, we expected that alignment to both parental genomes simultaneously (P1+P2) would result in haploid data, avoiding depth thresholding and greatly increasing genotyping efficiency for heterozygous germplasm.

**Core Ideas**

- Sequencing‐based genotyping of diploids suffers from undercalling of heterozygous genotypes.
- In interspecific hybrids, dual alignment to both parental genomes results in haploid genotypic datasets.
- Dual alignment reduces the proportion of “homeo‐SNPs” (fixed differences between nonallelic sites).

## 2 MATERIALS AND METHODS

### 2.1 Plant material

Pistachio interspecific hybrid rootstocks consisted of 768 seedlings derived from a single cross of *Pistacia atlantica × P. integerrima* provided by Foundation Plant Services and were sequenced in two Illumina HiSeq 4000 “SR100” lanes at the UC Davis Genome Center. Walnut interspecific hybrid rootstocks consisted of 228 clones derived from three crosses (*Juglans microcarpa* individuals DJUG31.01 (*n* = 105), DJUG31.09 (*n* = 113), and DJUG29.11 (*n* = 10) crossed with pollen from *J. regia* cv. Serr) (Ramasamy et al., 2021) and were sequenced in a single Illumina NextSeq 2000 “High75” lane. *Pistacia* parent trees are maintained by Foundation Plant Services, *J. microcarpa* mother trees are maintained at the National Clonal Germplasm Repository in Winters, CA, and *J. regia* cv. Serr and all clonal walnut rootstocks are maintained by the Walnut Improvement Program in Davis, CA.

### 2.2 Genotyping, imputation, and downsampling

Genotyping by sequencing (GBS) libraries were prepared as previously described (Poland et al., 2012) using simultaneous restriction ligation with HindIII‐HF, MseI, and T4 DNA Ligase (New England Biolabs). Following the TASSEL GBS pipelineV2 (Glaubitz et al., 2014), the Burrows‐Wheeler Aligner (Li & Durbin, 2009) was used to align 64 bp tags to reference assemblies for either the maternal parent species (P1), the paternal parent species (P2), or both maternal and paternal reference assemblies simultaneously (P1+P2). Reference assemblies for pistachio species, *J. microcarpa*, and *J. regia* were obtained from Palmer et al. (2023), Zhu et al. (2019), and Marrano et al. (2020), respectively. Only tags that aligned uniquely (MAPQ ≥ 20) were retained. The SNPQualityProfilerPlugin in TASSEL was used to remove candidate SNPs with low depth (log(depth) less than −1) and low inbreeding coefficient (*F* < −0.05 for P1 and P2 alignments; *F* < 0.9 for P1+P2 alignments) before variant calling with the ProductionSNPCallerPluginV2 in TASSEL. An inbreeding coefficient (*F*) of less than −0.05 represents excess heterozygosity relative to Hardy–Weinberg equilibrium; this filter was used to remove putative homeo‐SNPs from heterozygous data. A similar filter with *F* < 0.9 was used for the homozygous P1+P2 datasets in which no heterozygous genotypes are expected. Vcftools (Danecek et al., 2011) was used to remove indels, taxa with greater than 90% missing data, and for depth thresholding (minimum depth of five) of P1 and P2 datasets only. Imputation with Beagle 5.4 (Browning et al., 2018) was performed on unphased genotypes with no reference panel and a window size and walk speed of 12 and 4 Mb, respectively. Downsampling (50%) was performed using the reformat.sh command in bbmap (Bushnell, 2014).

## 3 RESULTS AND DISCUSSION

### 3.1 Effects of alignment strategy on heterozygosity and minor allele frequency

Three genotypic datasets each were generated for both *Pistacia* and *Juglans*, by aligning reads to the maternal genome only (P1), to the paternal genome only (P2), or to both maternal and paternal genomes simultaneously (P1+P2). Dual alignment to both parental genomes resulted in a higher proportion of reads aligning for hybrids of both genera (Table 1). SNPs with low coverage and excess heterozygosity were filtered from each dataset, and a depth threshold of five was applied to P1 and P2 datasets before imputation to minimize undercalling of heterozygotes. Results are summarized in Table 1. Note that *Juglans* and *Pistacia* have basic chromosome numbers of 16 and 15 and that dual alignment (P1+P2) results in alignment to 32 and 30 chromosomes respectively. The last column of Table 1 shows that in these low coverage datasets, depth thresholding with a minimum depth of five results in the removal of more than 80% of the raw data.

**TABLE 1:** Summary of *Juglans* and *Pistacia* datasets produced using three different alignment strategies

**Genus**	**Alignment strategy (% aligned)**	**Down‐sampling**	**Taxa**	**SNPs**	**Chromo‐somes**	**Average MAF**	**Heterozygote frequency**	**Data retained after depth thresholding (%)**
*Juglans*	*microcarpa* (P1; 63%)	None	228	114,965	16	0.163	0.066	18.3
		50%	226	113,380	16	0.147	0.048	10.2
	*regia* (P2; 55%)	None	228	83,355	16	0.191	0.093	16.4
		50%	228	83,515	16	0.168	0.064	10.7
	Dual (P1+P2; 76%)	None	224	65,517	32	0.29	0.003	100
		50%	222	64,607	32	0.28	0.003	100
*Pistacia*	*atlantica* (P1; 60%)	None	731	33,602	15	0.274	0.071	9.5
		50%	714	23,984	15	0.265	0.05	4.8
	*integerrima* (P2; 57%)	None	731	30,202	15	0.22	0.094	10.1
		50%	714	21,099	15	0.205	0.067	7.7
	Dual (P1+P2; 65%)	None	725	13,361	30	0.468	0.002	100
		50%	725	11,642	30	0.46	0.002	100

Abbreviations: MAF, minor allele frequency; SNP, single nucleotide polymorphism.

**FIGURE 2:**
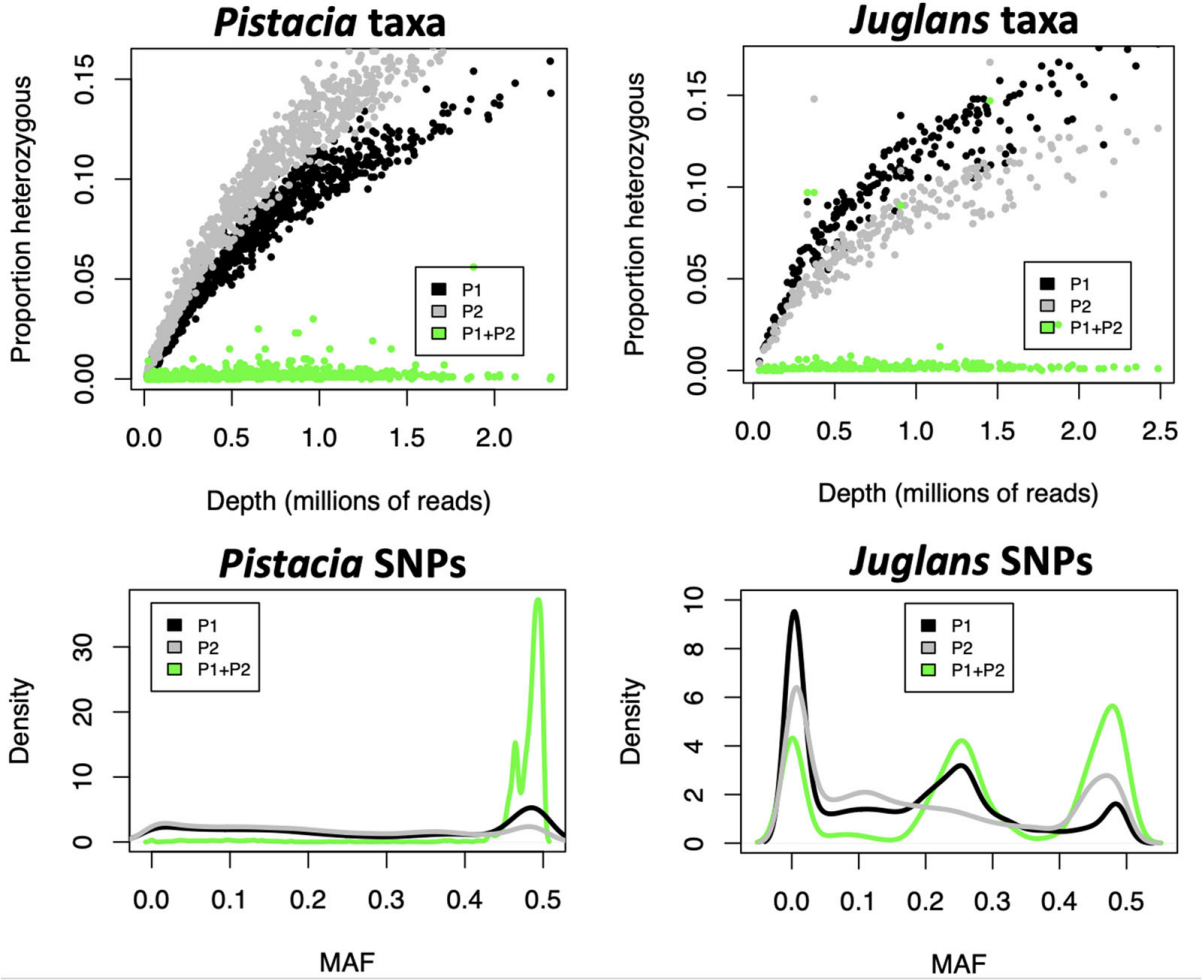
Effect of alignment strategy on the proportion of heterozygous genotypes (top) and minor allele frequency (MAF) distribution (bottom) in *Pistacia* and *Juglans*. Dual alignment to both parental genomes (P1+P2) results in effectively haploid datasets, whereas alignment to a single parental genome (P1 or P2) results in diploid datasets affected by undercalling of heterozygotes at low sequencing depth. MAF distributions for P1+P2 datasets more closely follow the expected distributions for a single family *(Pistacia)* and three families at frequencies of 0.48, 0.48, and 0.04 (*Juglans*).

Heterozygote frequencies in *Juglans* and *Pistacia* P1+P2 datasets are 0.003 and 0.002, respectively, 20–50× lower than in their corresponding P1 and P2 datasets (Table 1). The proportion of heterozygous genotypes in P1 and P2 datasets increases strongly with increasing read depth, whereas the proportion in P1+P2 datasets is not affected by read depth (Figure 2). The proportion of heterozygous genotypes is very close to zero for nearly all individuals in the P1+P2 datasets (top panels of Figure 2), and the few outliers with greater than 5% heterozygous genotypes likely result from cross‐contamination during DNA extraction or library preparation. Therefore, the dual alignment strategy for interspecific hybrids results in effective haploid datasets and enables simple detection and removal of cross‐contaminated samples.

The average minor allele frequency (MAF) in P1 and P2 datasets is approximately half of that in the corresponding P1+P2 datasets (Table 1). In the *Pistacia* P1+P2 dataset derived from a single cross, almost all SNPs have MAF values close to 0.5. MAF frequencies in the *Juglans* dataset are more variable since this population is derived from three families. Two of these families are at frequencies close to 0.5, and the third is at less than 0.05 frequency. In the *Juglans* P1+P2 dataset, three distinct peaks in the MAF distribution are observed close to 0.5, 0.25, and 0.05. The abundance of SNPs with MAF ∼0.125 in P1 and P2 (but not P1+P2) datasets likely indicates the segregation of diploid SNPs in one of the two more frequent Juglans crosses.

### 3.2 Shared and private SNPs across datasets

**FIGURE 3:**
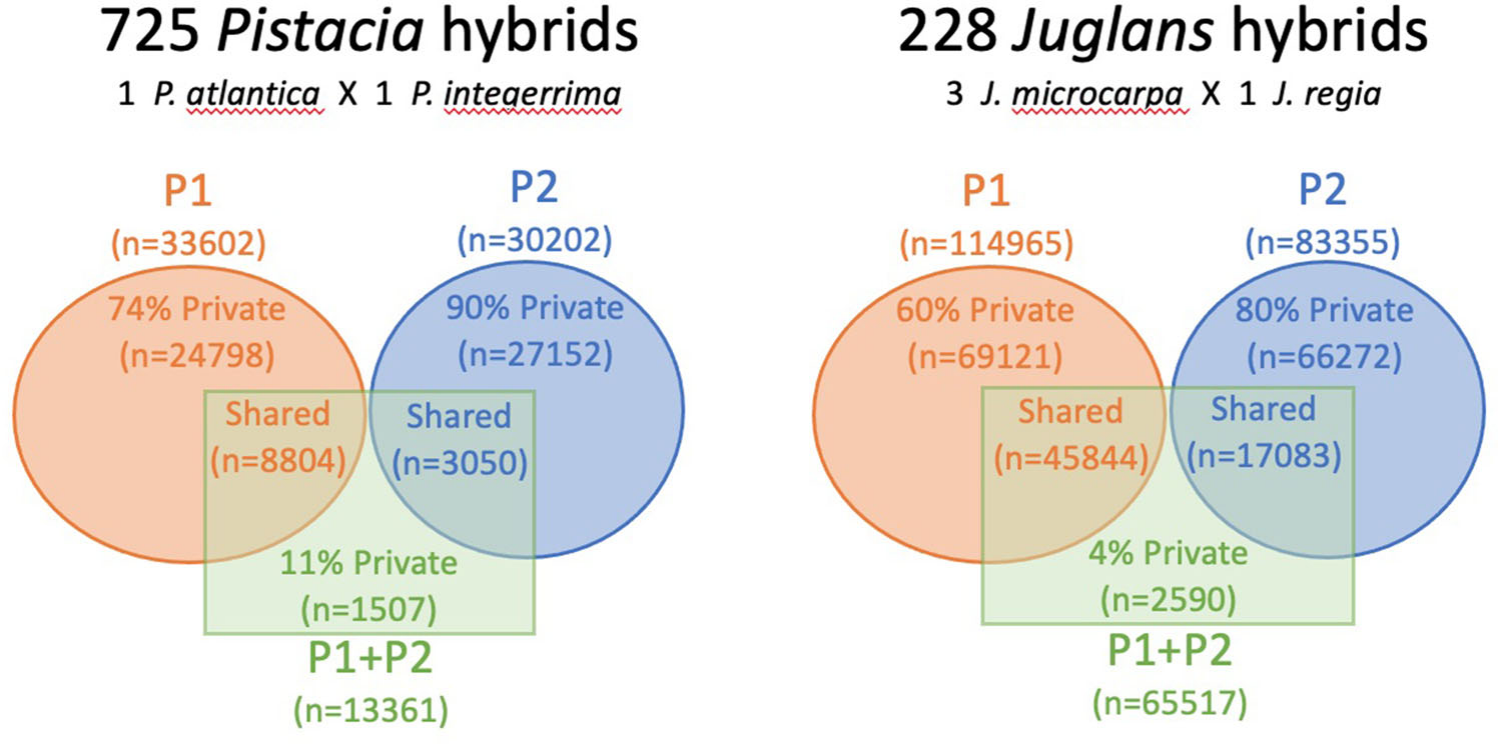
Shared and private single nucleotide polymorphisms (SNPs) in P1, P2, and P1+P2 datasets. Nearly all P1+P2 SNPs are shared with either the P1 or P2 dataset, whereas 60%–90% of P1 and P2 SNPs are private to that dataset. Numbers displayed in this figure are slightly higher than those shown in Figures 4 and 5 because downsampling reduces the number of SNPs available for comparison. There are no SNPs in common between P1 and P2 or between all three datasets.

Most SNPs in the P1+P2 datasets for each genus are shared with either the corresponding P1 or P2 dataset (Figure 3). However, the much larger P1 and P2 datasets are composed primarily (60%–90%) of private SNPs that are not present in the corresponding P1+P2 dataset. To investigate the hypothesis that these private SNPs are enriched with homeo‐SNPs, we compared Sequence Alignment Map (SAM) files resulting from alignment of unique reads to P1, P2, and P1+P2 genomes, and associated each SNP with its underlying reads in the SAM files based on position. A multi‐mapping index was defined for each SNP as the proportion of underlying unique reads that mapped to both parental genomes. Since homeo‐SNPs arise from multi‐mapped reads, we hypothesized that the reads underlying private SNPs would display a higher incidence of multi‐mapping. Indeed, the mean multi‐mapping index was ∼2X higher for private SNPs than for shared SNPs for both parents of both genera (Figure S1), suggesting that private SNPs are enriched with homeo‐SNPs.

### 3.3 Concordance between genotypes before and after downsampling

**FIGURE 4:**
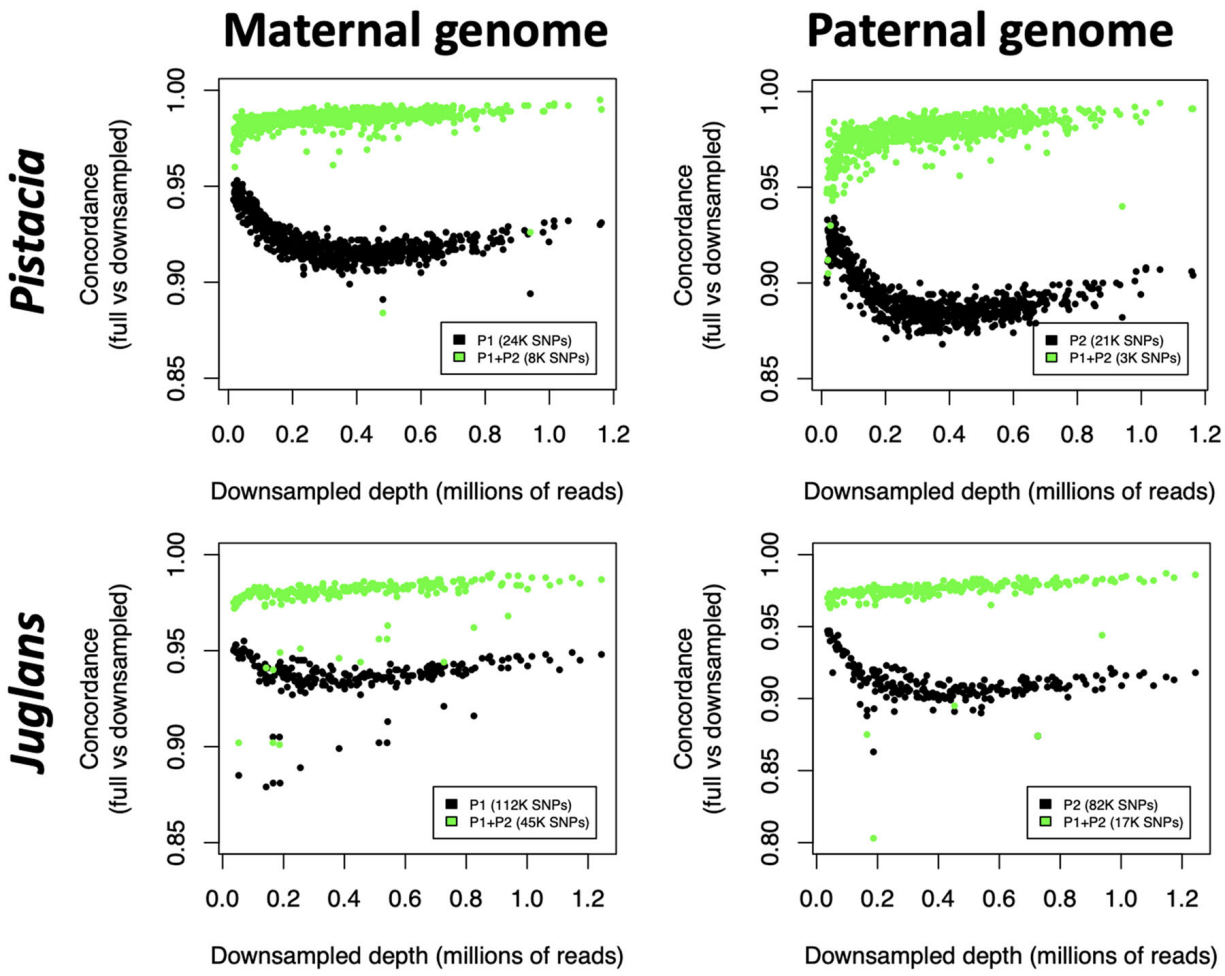
Effect of alignment strategy on robustness to reduced sequencing depth, as assessed by concordance of genotypes before and after 50% downsampling. Each point represents a hybrid, and the number of single nucleotide polymorphisms (SNPs) used to calculate concordance are shown in the legends. Separate P1+P2 concordance values were calculated for SNPs mapping to the maternal and paternal genomes.

To assess the robustness of each alignment strategy to reduced sequencing depth, raw Illumina files were downsampled by 50%, and genotype calls for each taxon from the imputed, downsampled datasets were compared to genotype calls from the full imputed datasets. Full and downsampled read counts for all *Pistacia* and *Juglans* taxa are shown in Tables S1 and S2. Concordance between full and downsampled datasets is much higher using dual alignment (P1+P2; Figure 4). Downsampling also results in a one‐third reduction in heterozygosity in P1 and P2 datasets but does not affect the proportion of heterozygous genotypes in P1+P2 datasets (Table 1). With increasing sequencing depth, concordance in P1+P2 datasets increases as expected, whereas concordance in P1 and P2 datasets is unexpectedly higher at low depth than at intermediate sequencing depth.

### 3.4 Concordance of shared and private SNPs in P1 and P2 datasets

**FIGURE 5:**
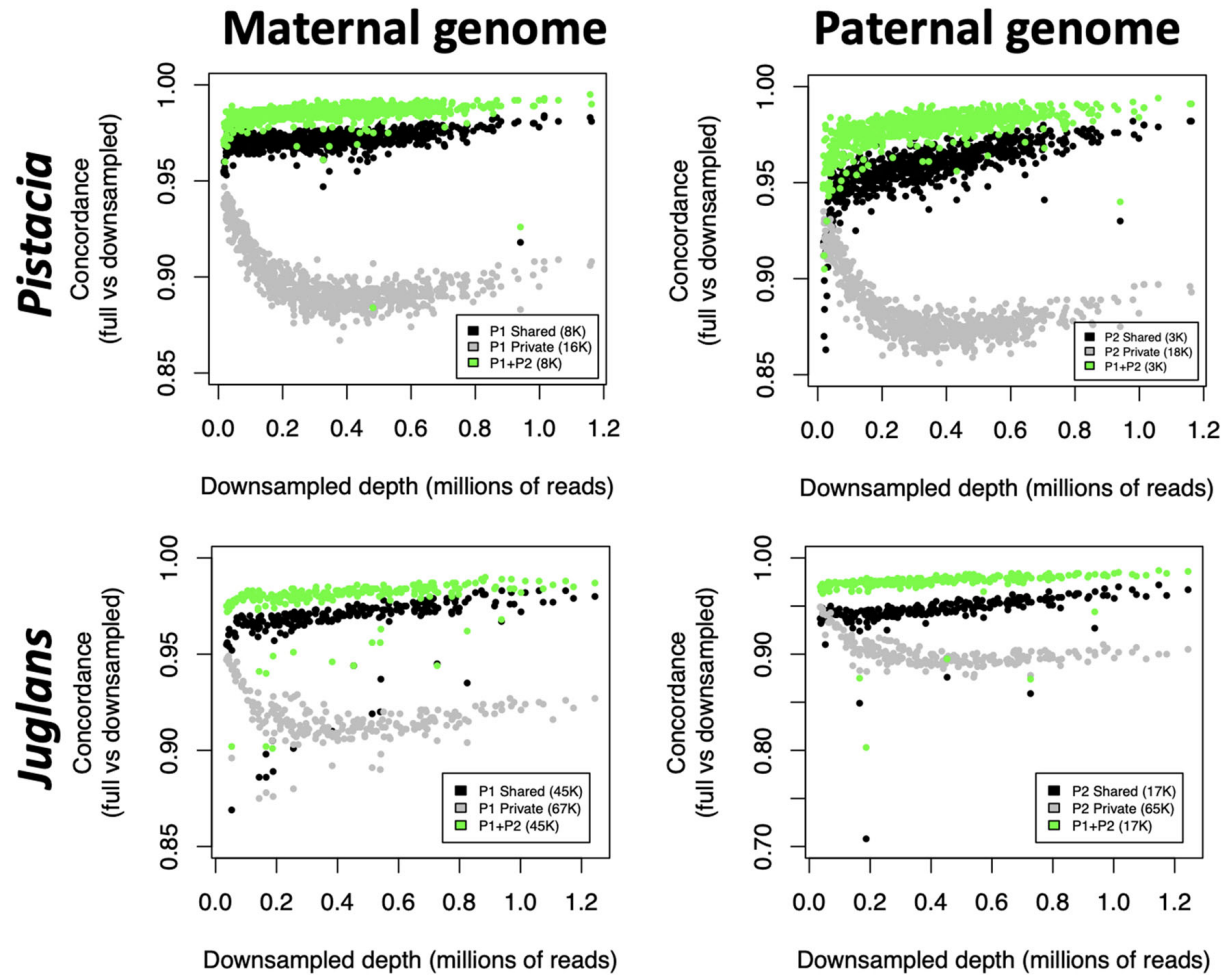
Differences in concordance between shared and private single nucleotide polymorphisms (SNPs) in P1 and P2 datasets. P1+P2 concordance values shown in green are the same as in Figure 4, but P1 and P2 concordance values have been calculated separately for shared (black) and private (gray) SNPs. Note that concordance values for shared SNPs and P1+P2 SNPs in each plot are calculated using exactly the same set of loci, but these loci show haploid segregation in P1+P2 SNPs and mostly diploid segregation in P1/P2 shared SNPs.

P1 and P2 SNPs that are shared with the corresponding P1+P2 dataset have much higher levels of concordance than private SNPs in the P1 and P2 datasets (Figure 5). In addition, these shared SNPs show the expected increase in concordance with increasing sequence depth, similar to P1+P2 SNPs, whereas private SNPs show the unexpected decrease in concordance at intermediate sequencing depth previously noted in Figure 4. This phenomenon could be due to enrichment with homeo‐SNPs, which are truly heterozygous in all individuals but may appear as homozygous when sequencing depth is limited. One limitation of our reduced representation library preparation protocol is that alleles underlying a locus may have different fragment sizes, leading to amplification bias against larger fragments (Davey et al., 2011). In samples with very low sequencing depth, under‐represented reads from the larger fragment may be absent, leading to higher concordance at low depth in the private SNPs.

### 3.5 Alignment to a single parental genome is ineffective at resolving population structure in the other parent

**FIGURE 6:**
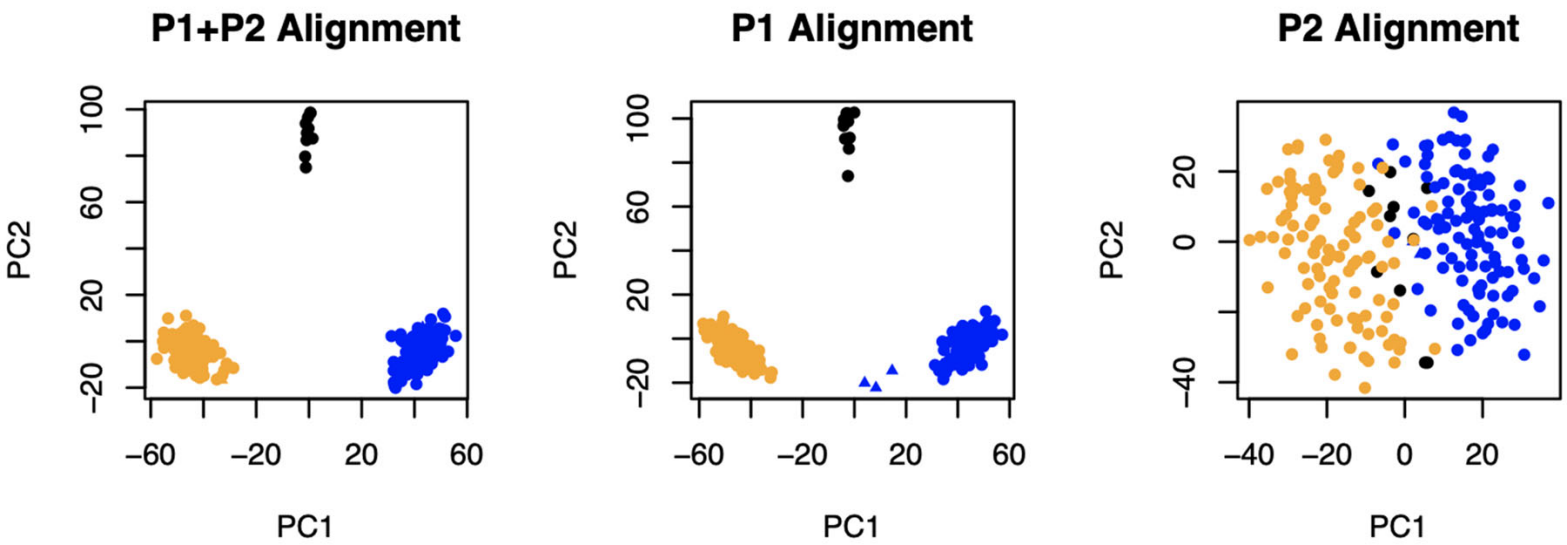
Principal component analysis (PCA) to resolve population structure in *Juglans* hybrids using different alignment methods. Orange (*n* = 105), blue (*n* = 113), and black (*n* = 10) dots represent hybrids derived from three different maternal parents; all hybrids share the same paternal parent. Alignment to both parental genomes (P1+P2) or the maternal genome (P1) resolves this structure, whereas alignment to the paternal genome (P2) does not. Three hybrids sequenced at very low depth (<30,000 reads) are shown as blue triangles. Alignment to both parents results in effectively haploid data that are much more robust to low sequencing depth.

Principal component analysis (PCA) was applied to P1, P2, and P1+P2 datasets to model population structure in the *Juglans* population of hybrids, which consists of three families derived from three maternal parents and a single paternal parent (Figure 6). Although P1+P2 and P1 datasets were effective at resolving this population structure, the P2 dataset was not. To interpret this result, we note that the *Juglans* P2 dataset contains far fewer SNPs at MAF ∼0.25 (Figure 2), the frequency we would expect for a diploid SNP showing a fixed difference between the two common maternal parents. It is possible that a mapping quality threshold less stringent than the one used in this study (MAPQ ≥ 20) might have been effective in retaining more genetic structure in the nonaligned parent dataset. Although the PCA plots for P1 and P1+P2 look overall quite similar, the three samples with the fewest sequencing reads (shown as blue triangles in Figure 6) drift towards the origin in the P1 dataset but not the P1+P2 dataset, suggesting the latter is more robust to low sequencing depth.

### 3.6 Future work and similarity with other methods

Inbred or haploid genotypic datasets enjoy huge quality advantages over heterozygous datasets at comparable levels of sequencing depth. This study used a minimum depth threshold of five for P1 and P2 datasets (assuming no amplification bias, 93.75% of heterozygous sites with a depth of exactly five would be called correctly) which resulted in ∼80%–90% of the raw data being discarded (Table 1). The luxury of relaxing or removing depth thresholds in inbred datasets results in retention of much more data, and summarizing heterozygosity by taxa or by SNP in inbred datasets simplifies the removal of cross‐contaminated DNA samples and homeo‐SNPs respectively. In this study, dual alignment of reads from interspecific hybrids to both parental genomes (P1+P2) resulted in effectively inbred datasets that enabled more rigorous quality control, displayed higher concordance following downsampling, and provided more robust estimation of population structure compared to standard alignment against a single reference genome. Although this study used Beagle imputation for purposes of comparing different alignment strategies, datasets resulting from dual alignment could also be imputed using FSFHap, an imputation method designed for inbred populations (Swarts et al., 2014), whereas P1 and P2 datasets could not. The practical conclusion of this study is that dual alignment allows interspecific hybrids to be genotyped and imputed as efficiently and inexpensively as inbreds.

The divergence between parental genomes in this study is estimated at 38 million years for *Pistacia* (*P. atlantica* vs. *P. integerrima*) (Xie et al., 2014) and 45 million years for *Juglans* (*J. microcarpa* vs. *J. regia*) (Stevens et al., 2018). This study used 90 bp Illumina reads trimmed to 64 bp for speedier processing through the TASSEL GBS pipeline (Glaubitz et al., 2014), of which 65% and 76% mapped uniquely to the *Pistacia* and *Juglans* P1+P2 genomes, respectively. Longer reads could be used to apply this strategy to hybrids with lower divergence, and perhaps even hybrids between heterotic groups within a species. Alternatively, strategies that make use of a “pan‐genome,” including the Practical Haplotype Graph (Bradbury et al., 2022), may achieve a similar result by including enough representative reference contigs to ensure that all reads align to a homologous (nonhomeologous) sequence. The strategy described here might also be applied to transcriptome data of hybrids to investigate allele‐specific or species‐specific patterns of expression and co‐expression.

## AUTHOR CONTRIBUTIONS

**Pat J. Brown**: Conceptualization, Formal analysis, Writing‐original draft.

## CONFLICT OF INTEREST STATEMENT

The author declares no conflicts of interest.

## FUNDING INFORMATION

USDA NIFA‐SCRI grant no. 2018‐51181‐28437, the California Walnut Board, and the California Pistachio Research Board.

## Supporting information

Figure S1. Distribution of the multi‐mapping index for private and shared SNPs in the P1 and P2 datasets of *Pistacia* and *Juglans*. The multi‐mapping index is defined for each SNP as the proportion of unique reads underlying that SNP that map to both parental genomes.

Supplement Material

Supplement Material

## Data Availability

Raw sequence data for this project have been submitted to the NCBI SRA under BioProject PRJNA909784. Twelve imputed VCF files representing two genera, three alignment strategies, and two levels of downsampling are included as Supporting Information.
